# The Prevalence of Glaucoma in the Jirel Ethnic Group of Nepal

**DOI:** 10.3389/fopht.2022.824904

**Published:** 2022-06-27

**Authors:** Sarah Miller, Nicholas B. Blackburn, Matthew Johnson, Sandra Laston, Janardan Subedi, Jac C. Charlesworth, John Blangero, Bradford Towne, Suman S. Thapa, Sarah Williams-Blangero

**Affiliations:** ^1^Department of Family Medicine, School of Medicine, University of California Davis, Sacramento, CA, United States; ^2^Menzies Institute for Medical Research, University of Tasmania, Hobart, TAS, Australia; ^3^South Texas Diabetes and Obesity Institute, University of Texas Rio Grande Valley, Brownsville, TX, United States; ^4^Department of Human Genetics, School of Medicine, University of Texas Rio Grande Valley, Brownsville, TX, United States; ^5^Department of Sociology and Gerontology, College of Arts and Sciences, Miami University, Oxford, OH, United States; ^6^Department of Community Health, School of Medicine, Wright State University, Dayton, OH, United States; ^7^Tilganga Institute of Ophthalmology, Kathmandu, Nepal

**Keywords:** glaucoma, ocular epidemiology, Asia, eye health, eye disease, Nepal

## Abstract

Glaucoma is one of the leading causes of blindness worldwide with individuals in Asia disproportionately affected. Using a cross-sectional study design as part of the Jiri Eye Study, we assessed the prevalence of glaucoma in the Jirel population of Nepal and provide new information on the occurrence of glaucoma in south central Asia. Over a four-year period, 2,042 members of the Jirel population, aged 18 years and older, underwent a detailed ocular examination. Glaucoma was diagnosed using the International Society of Geographical and Epidemiological Ophthalmology criteria. The mean (SD) age at exam was 42.3 (16.7) years and 54.1% of the sample was female. In the total sample, the mean (SD) intraocular pressure (IOP) and vertical cup-to-disc ratio (VCDR) was 14.55 (2.42) mmHg and 0.31 (0.15), respectively. The 97.5^th^ and 99.5^th^ percentile for IOP and VCDR was 20 mmHg and 22 mmHg, and 0.7 and 0.8, respectively. The overall prevalence of glaucoma in the population was 2.30% (n = 47). Of these 47 individuals, 37 (78.7%) had primary open angle glaucoma, 6 (12.8%) had primary angle closure glaucoma, and 4 (8.5%) had secondary glaucoma. There was a significant (p = 5.86×10^−6^) increase in the prevalence of glaucoma with increasing age overall and across glaucoma subtypes. Six individuals with glaucoma (12.8%) were blind in at least one eye. Of the individuals with glaucoma, 93.6% were previously undiagnosed. In individuals aged 40 years or older (n = 1057, 51.4% female), the mean (SD) IOP and VCDR was 14.39 (2.63) mmHg and 0.34 (0.16), respectively, and glaucoma prevalence was 4.16% (n = 44). The prevalence of glaucoma and undiagnosed disease is high in the Jirel population of Nepal. This study will inform strategies to minimize glaucoma-associated burden in Nepal.

## Introduction

Glaucoma is the second leading cause of blindness worldwide despite the availability of treatments proven to minimize vision loss due to glaucoma (1–4). It is estimated that approximately 64.3 million individuals world-wide have glaucoma, with individuals in Asia disproportionately affected (1, 5). Early detection of glaucoma is vital to help arrest disease progression and prevent vision loss, since the prognosis for cases that do not receive prompt treatment is poor (6, 7). The number of individuals with glaucoma is projected to reach 111.8 million by 2040, with the largest increases in prevalence being seen in Asia and Africa (5).

The prevalence of glaucoma varies across ethnic populations. The overall prevalence of glaucoma in Asia has been found to range between 2.1% and 5.0% (8–17). In south central Asia (India, Iran, Nepal, and Sri Lanka), the prevalence of glaucoma has been found to be 3.5%, and primary open-angle glaucoma (POAG) is the most common form (17). There has been little research on the prevalence of glaucoma in Nepal (18–22). One recent population-based study in Nepal found the overall prevalence of glaucoma to be 1.80% in people over 40 years old, with the prevalence of POAG higher than primary angle closure glaucoma (PACG), 1.24% and 0.39%, respectively (18), which is consistent with the findings of previous studies (20–22).

Of the available Nepalese studies, the majority have been conducted in hospital-based settings (20–22). Given the relatively high prevalence of glaucoma and the ethnic diversity within Nepal, it is important to study the prevalence of glaucoma in different regions and among different ethnic groups of the country. This study aimed to determine the prevalence of glaucoma in the Jirel population, a small ethnic group residing in the Dolakha District of eastern Nepal.

## Materials and Methods

### Study Population

The Jirel population has been the focus of a broad range of anthropological and biomedical studies for more than three decades. These studies have included anthropological investigations (23), assessments of population structure (24), studies of genetic susceptibility to parasitic worm infection (25), and studies of the genetics of growth and development (26). Current research in the Jirel population is focused on the genetic epidemiology of ocular traits and disorders as part of the Jiri Eye Study (JES) (27). The long history of genetic research conducted with the Jirels has resulted in the availability of extensive genealogical information for the population. All individuals (n ~3,000) who have participated in previous studies and the current ocular study belong to a single extended pedigree, which makes this cohort an extremely powerful resource for genetic epidemiological studies (28). The catchment region for this longitudinal population-based study included three Village Development Committees/Municipalities (Jiri, Jungu, and Chhetrapa) located in Dolokha District. According to the 2011 Nepal National Census (29), there were 4,239 Jirels residing in the study area in 2011.

### Subject Recruitment

JES inclusion criteria required individuals to be in good health, at least 18 years of age, and a member of the Jirel population. Subject recruitment and examination activities took place during semi-annual visits to the field research site in Jiri, Nepal across a four-year period (2015 to 2018). Prior to each field session, local recruitment staff contacted potential study participants by house in person to explain the purpose and benefits of the study, provide them with a copy of the Nepali-translated consent form (the consent form was read to individuals who were illiterate), obtain verbal agreement to participate in the study, and arrange an appointment date and time to attend the field research clinic that coincided with a forthcoming field site visit. Upon arrival at the clinic on their designated day, individuals were given time to re-read the consent form or have it re-read to them and to ask any questions they might have. Informed consent was documented by a witnessed signature or thumb print. All study procedures were conducted in accordance with a protocol approved by the University of Texas Rio Grande Valley Institutional Review Board and the Nepal Health Research Council. Procedures were in accordance with the Declaration of Helsinki.

### Clinical Examination

A comprehensive ophthalmic examination was performed for all JES participants. Unilateral distance and near visual acuities (VAs) – both presenting and best corrected – were measured using a LogMAR tumbling E chart placed at 4 meters. If a study participant was unable to identify the direction of the largest ‘E’ optotypes, perception of hand movement or light (if hand movements could not be perceived) were assessed. LogMAR unit measurements were converted to decimal acuity for analyses according to the formula: decimal acuity = 10^−LogMAR^, as per Holladay, 1997 (30).

Slit lamp biomicroscopy (BD900, Haag-Streit International, Bern, Switzerland) of the anterior and posterior segments was conducted to identify ocular abnormalities, specifically, ischemic sequelae of previous acute angle closure, secondary glaucoma, or evidence of previous glaucoma surgery. The van Herick method was used to grade the depth of the peripheral anterior chamber. Intraocular pressure (IOP) was measured with a Goldmann applanation tonometer (AT900, Haag-Streit International) after administration of an anesthetizing solution (proparacaine 0.5%) and staining of the cornea with a dry strip of fluorescein. Gonioscopy was performed using a 4-mirror gonioscope (Carl Zeiss AG, Oberkochen, Germany) in ambient light with a shortened slit beam that did not fall on the pupil; the iridocorneal angle was graded using the Shafer system.

For all individuals with a best corrected VA of 0.30 or greater, a 24-2 visual field test was performed using a Humphrey HFA II 745i Visual Field Analyzer (Carl Zeiss, Bangalore, India).

A mydriatic examination was conducted in all participants unless contraindicated due to risk of angle closure. Fundus examination was performed with a 90-diopter lens (Volk Optical Inc., Mentor, OH, USA) to evaluate the optic disc and macula. Vertical cup-to-disc ratios (VCDR) were measured using an eye piece graticule and recorded in units of 0.05 (Haag-Streit International).

### Glaucoma Diagnosis

Glaucoma cases were defined based on the structural and functional evidence recommended for cross sectional population-based research, according to the International Society of Geographic and Epidemiologic Ophthalmology (ISGEO) scheme (**Table 1**) (31). Briefly, the distribution of VCDR from non-glaucomatous subjects with a normal visual field result for both eyes was calculated, where normal visual fields were defined upon application of the ISGEO scheme to this population’s distribution. Optic discs were considered normal if the VCDR of one or both discs, or disc asymmetry was less than the 97.5^th^ percentile of this distribution. A glaucomatous visual field defect was present when (1) the hemifield test result was outside normal limits, and (2) a cluster of three or more non-edge, contiguous points, not crossing the horizontal meridian, with a probability of less than 5% of an age-matched normal control on the pattern deviation was noted. In the presence of an open anterior chamber angle (≥ 180° of the pigmented trabecular meshwork), a JES participant was assigned a diagnosis of POAG if one or both eyes had evidence of glaucoma (**Table 1**), unless there was evidence of retinal or optic nerve disease, pseudoexfoliation, trauma or pigment dispersion. If any of these latter observations were made, a diagnosis of secondary open angle glaucoma was assigned. An occludable angle was diagnosed if the posterior trabecular meshwork was not observed for an angle > 180° on non-indentation gonioscopy. PACG was defined as an eye with an occludable angle, peripheral anterior synechiae and/or elevated IOP with evidence of glaucoma. JES participants with an occludable angle but no evidence of glaucoma and a normal IOP were diagnosed as primary angle closure suspects (PACS).

**Table 1:** Glaucoma diagnosis categories^a^.

**Category 1:** Structural and functional evidence.	Eyes with VCDR or VCDR asymmetry ≥ 97.5^th^ percentile for the normal population combined with a visual field defect consistent with glaucoma.
**Category 2:** Advanced structural damage where reliable field testing is not possible.	Eyes with VCDR or VCDR asymmetry ≥ 99.5^th^ percentile for the normal population.
**Category 3:** Optic disc not seen because of media opacity.	Visual acuity < 1.30 and IOP > 99.5^th^ percentile, or Visual acuity < 1.30 and the eye shows evidence of glaucoma filtering surgery.

a In diagnosing category 1 or 2 glaucoma, it was required that there should be no alternative explanation for CDR findings (dysplastic disc or marked anisometropia) or the visual field defect (retinal vascular disease, macular degeneration, or cerebrovascular disease).

VCDR, vertical cup-to-disc ratio; IOP, intraocular pressure.

### Visual Impairment Definition

Definitions of visual impairment (VI) (best corrected VA) are derived from the International Classification of Diseases, 11^th^ edition (32). Here, we define no VI as VA ≥ 0.5, mild VI as VA of < 0.50 but ≥ 0.30, moderate VI as VA < 0.30 but ≥ 0.10, severe VI as VA < 0.10 but ≥ 0.05, and blindness as VA < 0.05 (includes no hand movement or light perception) with best correction or a visual field < 10° from fixation.

### Statistical Analysis

Comparisons between unaffected individuals and individuals affected with glaucoma subtypes, as well as glaucoma subtype associations with age, sex and their interactions (age^2^, age × sex, age^2^ × sex) were calculated in variance components models in SOLAR (v8.1.1) to account for the non-independence of the related individuals in this cohort (33). Statistical analysis for dichotomous affection status employed a probit threshold model in which the underlying latent risk distribution was assumed to be normally distributed. The model is very similar to a logistic model but is better suited to allow for residual non-independence amongst genetically related individuals. SOLAR was used to analyze IOP and VCDR (continuous variables) for their relationships to glaucoma and glaucoma subtypes, controlling for age, sex and their interactions. Descriptive statistical analyses were performed using R 3.5.0 (RStudio, Inc., Boston, MA).

## Results

There were 2042 individuals with complete physical exam data for analysis, 2016 with complete IOP measurements and 2036 with VCDR measurements. The mean age of the study population was 42.3 years (SD, 16.7 years), and more women were examined (54.1%) than men. The mean (SD) of IOP and VCDR were 14.55 (2.42) mmHg and 0.31 (0.15), respectively. The 97.5^th^ and 99.5^th^ percentile for IOP were 20 mmHg and 22 mmHg, respectively, and 0.7 and 0.8 for VCDR, respectively. There were 47 subjects with glaucoma, of whom 37 (78.7%) had POAG, 6 (12.8%) had PACG, and 4 (8.5%) had secondary glaucoma. Additionally, 77 individuals were assigned PACS. For individuals aged 40 years and older (“40+ subgroup”; n = 1057, 51.4% female), the mean (SD) of IOP and VCDR was 14.39 (2.63) mmHg and 0.34 (0.16), respectively.

The overall prevalence of glaucoma was 2.30% (**Table 2**). Crude overall prevalence of POAG and PACG was 1.81% and 0.29%, respectively. After accounting for the non-independence of related individuals there was a significant (p = 5.86×10^−6^, β=0.04) increase in prevalence of glaucoma with increasing age overall and across glaucoma subtypes. In the 40+ subgroup, the overall prevalence of glaucoma was 4.16% with a prevalence of POAG and PACG of 3.41% and 0.57%, respectively. The prevalence of glaucoma overall was not related to sex (p = 0.41) and a sex specific age effect was not observed (age × sex, p = 0.78), indicating that the prevalence of glaucoma increased with age regardless of sex. These findings were consistent across glaucoma subtypes (POAG, PACG).

**Table 2:** Prevalence of glaucoma in the Jirel population by age and sex.

Age, yrs	All Persons		Male		Female	
	N (%)	Affected (%)	N (%)	Affected (%)	N (%)	Affected (%)
All Glaucoma^a^						
<40	985 (48.2)	3 (0.30)	423 (45.1)	1 (0.24)	562 (50.9)	2 (0.36)
40-49	370 (18.1)	8 (2.16)	171 (18.2)	4 (2.34)	199 (18.0)	4 (2.01)
50-59	317 (15.5)	12 (3.79)	163 (17.4)	8 (4.91)	154 (13.9)	4 (2.6)
60-69	232 (11.4)	10 (4.31)	116 (12.4)	7 (6.03)	116 (10.8)	3 (2.59)
70-79	114 (5.58)	13 (11.4)	52 (5.55)	6 (11.5)	62 (5.61)	7 (11.3)
≥80	24 (1.18)	1 (4.17)	12 (1.28)	0 (0)	12 (10.9)	1 (8.33)
Crude	2042	47 (2.30)	937	26 (2.77)	1105	21 (1.90)
POAG						
<40	985 (48.2)	1 (0.10)	423 (45.1)	0 (0)	562 (50.9)	1 (0.18)
40-49	370 (18.1)	8 (2.16)	171 (18.2)	4 (2.34)	199 (18.0)	4 (2.01)
50-59	317 (15.5)	9 (2.84)	163 (17.4)	5 (3.07)	154 (13.9)	4 (2.6)
60-69	232 (11.4)	7 (3.02)	116 (12.4)	5 (4.31)	116 (10.8)	2 (1.72)
70-79	114 (5.58)	11 (9.65)	52 (5.55)	6 (11.5)	62 (5.61)	5 (8.06)
≥80	24 (1.18)	1 (4.17)	12 (1.28)	0 (0)	12 (10.9)	1 (8.33)
Crude	2042	37 (1.81)	937	20 (2.13)	1105	17 (1.54)
PACG						
<40	985 (48.2)	0 (0)	423 (45.1)	0 (0)	562 (50.9)	0 (0)
40-49	370 (18.1)	0 (0)	171 (18.2)	0 (0)	199 (18.0)	0 (0)
50-59	317 (15.5)	2 (0.63)	163 (17.4)	2 (1.23)	154 (13.9)	0 (0)
60-69	232 (11.4)	2 (0.86)	116 (12.4)	1 (0.86)	116 (10.8)	1 (0.86)
70-79	114 (5.58)	2 (1.75)	52 (5.55)	0 (0)	62 (5.61)	2 (3.23)
≥80	24 (1.18)	0 (0)	12 (1.28)	0 (0)	12 (10.9)	0 (0)
Crude	2042	6 (0.29)	937	3 (0.32)	1105	3 (0.27)

a Includes 4 secondary glaucoma cases: 3 males (<40 yrs, 50-59 yrs, 60-69 yrs), 1 female (<40 yrs); crude prevalence, 0.2%. POAG, primary open-angle glaucoma; PACG, primary angle closure glaucoma.

In **Table 3**, we present the demographics of glaucoma cases overall and across glaucoma subtypes. While not statistically significant, more males than females were diagnosed with glaucoma overall and across glaucoma subtypes. The mean age of glaucoma patients was 60.4 years with secondary glaucoma patients having the lowest mean age (46 years) and PACG patients having the highest (64.8 years). For glaucoma patients, the average IOP was 16.2 mmHg and the average VCDR was 0.6. Among subjects diagnosed with glaucoma in this study, 93.6% had not been previously diagnosed. Of those that had been previously diagnosed (n=3), one was currently taking glaucoma medication.

**Table 3:** Demographics of glaucoma cases in the Jirel population.

	All (n)	Male (n)	Female (n)	Male : Female ratio	Mean age, yrs	IOP (mm Hg)	VCDR	Previously diagnosed (%)
POAG	37	20	17	1.18	61.22	15.63	0.61	2 (5.41)
PACG	6	3	3	1.0	64.83	18.8	0.56	1 (16.7)
Secondary glaucoma	4	3	1	3.0	46	18.25	0.53	0 (0)
Total	47	26	21	1.24	60.38	16.23	0.6	3 (6.38)

POAG, primary open-angle glaucoma; PACG, primary angle closure glaucoma; IOP, intraocular pressure; VCDR, vertical cup-to-disc ratio.

In **Table 4**, we present the distribution of age and sex, and IOP and VCDR measurements in the Jirels without glaucoma and those diagnosed with POAG or PACG. The mean age of individuals with POAG (61.2 yrs) or PACG (64.8 yrs) was significantly higher (p = 3.39×10^−5^ and 8.10×10^−4^ respectively) than in unaffected individuals (41.4 yrs). As expected, IOP and VCDR measurements were significantly increased in glaucoma cases. IOP was significantly higher in both the PACG (18.8 vs. 14.5 mm Hg, p = 5.27×10^−5^) group and the POAG group (15.6 vs 14.5, p = 0.002) compared to individuals without disease. POAG cases had IOP that was 0.52 SDU (standard deviation units) higher, while PACG cases had IOP that was 1.76 SDU higher than unaffected individuals. The VCDR in individuals with POAG (0.61 vs. 0.3) was significantly higher (p = 5.82×10^−16^), with a 1.17 SDU increase in comparison to unaffected individuals. The VCDR in individuals with PACG (0.56) was similar to individuals with POAG with a 1.01 SDU increase in comparison to unaffected individuals (p = 0.018).

**Table 4:** Distribution of age, sex, IOP and VCDR in the Jirel population with and without glaucoma.

Characteristic	Unaffected (SD)	POAG (SD)	p-value	PACG (SD)	p-value
Age, yrs	41.4 (16.5)	61.2 (13.2)	3.39 × 10^−5^	64.8 (10.7)	8.10 × 10^−4^
Sex, Male/Female	899/1019	20/17	0.98	3/3	0.79
IOP	14.5 (2.3)	15.6 (3.7)	0.002	18.8 (3.0)	3.67 × 10^−5^
VCDR	0.3 (0.14)	0.61 (0.25)	5.82 × 10^−16^	0.56 (0.31)	0.008

POAG, primary open-angle glaucoma; PACG, primary angle closure glaucoma; IOP, intraocular pressure; VCDR, vertical cup-to-disc ratio.

In **Table 5**, we present the VA of glaucoma cases (n = 47) in the Jirel population by age, sex and glaucoma sub-type. Six subjects (12.8%) with glaucoma were blind in at least one eye and 3 of these subjects were blind in both eyes. six (12.8%) had moderate VI (POAG: 5, PACG: 1), four (8.5%) had mild VI (POAG: 4) and 34 (72.3%) had no to mild VI (POAG: 26, PACG: 4, Secondary glaucoma: 4). No glaucoma cases had severe VI. Of the three glaucoma cases who were bilaterally blind, two had bilateral POAG and one unilateral PACG. Of the three unilaterally blind glaucoma cases, one had secondary glaucoma in their blind eye and the remaining two did not have glaucoma in their blind eye but POAG with moderate VI in their seeing eye, which may suggest the presence of additional factors in their blindness.

**Table 5:** Impaired visual acuity of glaucoma cases in the Jirel population.

	N	Impaired Visual Acuity^a^			
		No VI	Mild VI	Moderate VI	Blind
Age Group (yrs)					
<40	3	3	0	0	0
40-49	8	8	0	0	0
50-59	12	11	0	0	1
60-69	10	7	2	1	0
70-79	13	5	2	5	1
≥80	1	0	0	0	1
Sex					
Male	26	21	1	3	1
Female	21	13	3	3	2
Types of Glaucoma					
POAG	37	26	4	5	2
PACG	6	4	0	1	1
Secondary glaucoma	4	4	0	0	0
Total	47	34	4	6	3

a See methods for visual acuity definitions.

POAG, primary open-angle glaucoma; PACG, primary angle closure glaucoma; VI, visual impairment.

## Discussion

Glaucoma is an ocular disease of global health significance. In south central Asian countries, the number of individuals with glaucoma is projected to increase by approximately 93% in the next 20 years (17). In Nepal, glaucoma is a growing public health issue and studies estimating its prevalence provides valuable information to promote eye health and disease awareness programs with a view to lessen its burden (18).

The overall prevalence of glaucoma in this study is higher than that seen in the Bhaktapur Glaucoma Study (BGS) (JES: 2.30%, BGS: 1.80%) (18). When comparing glaucoma subtypes between this study and the BGS, POAG was higher in this study (JES: 1.81%, BGS: 1.24%) and PACG was lower (JES: 0.29%, BGS: 0.39%), but secondary glaucoma was similar (JES: 0.2%, BGS: 0.15%) (18). Among the JES POAG subjects, 27 (73%) were diagnosed as having normal tension glaucoma (NTG). Proportionally, more patients had NTG out of all glaucoma cases in this study than previously reported in Nepal (JES: 57.4%, Lahan: 14.6%) (20), or other south central Asian counties (Iran: 34.5%) (12). NTG cases, however, may be overestimated as the maximum diurnal IOP level may have been overlooked due to a single time point measurement during daylight hours in our study. Additionally, Jiri is approximately 2500 m above sea level, and some studies conducted at similar or higher altitudes (2800-5850m) have shown that while IOP may initially increase (34), it may decrease with continued exposure to higher atmospheric pressure (35). The regulation of the body *via* decreased episcleral venous pressure due to lower temperatures at high altitude has been shown to lower IOP and may also play a role in the high proportion of NTG seen in this population (36). Therefore, more effective screening in the Jirels and other high-altitude populations may be achieved by using other structural and functional ophthalmologic measurements, such as retinal nerve fiber layer thickness and VCDR, in addition to IOP (37–40).

For JES individuals in the 40+ subgroup, the prevalence of overall glaucoma is approximately 2.3 times higher than that previously reported in Nepal (JES: 4.16%, BGS: 1.80%) (18),. Similarly, the prevalence of POAG in this 40+ subgroup was approximately 2.7 times higher than that previously reported (JES: 3.41%, BGS: 1.24%); PACG was approximately 1.5 times higher in the JES 40+ subgroup (JES: 0.57%, BGS: 0.39%) (18). These findings highlight the significance of the burden of glaucoma in the Jirel population and indicate a need to further investigate the driving factors behind the high prevalence of disease.

Prevalence of glaucoma was found to increase with age across all subtypes indicating an increasing public health problem as individual’s longevity increases in Nepal (41). Also, we note that the rate of undiagnosed glaucoma in the Jirels is very high (Overall: 93.62%, POAG: 95%, PACG: 83%), which is similar to other regions of Nepal (85.33%) (18), and south central Asia (82.8-94.1%) (8–12, 42). These metrics highlight the need to promote glaucoma awareness across Nepal, and Asia in general, and prioritize the development of strategies to improve glaucoma screening programs. This is of greater importance in remote regions where the ratio of ophthalmic care facilities/trained medical staff to local populations may be low or moderate. Moreover, slow (glaucoma) disease progression may also contribute to the low rate of prior diagnoses and lack of awareness. The use of mobile imaging tools rather than traditional table-top cameras to assess, for example, VCDR and visual field loss are positive steps in the effort to screen for glaucoma in remote settings (43, 44). These technologies have been shown to increase identification of posterior segment pathologies, such as glaucoma, and address barriers to the delivery of care by being affordable, portable, and easy to use (45, 46).

This study found that 12.8% of all glaucoma patients were blind in at least one eye. Higher rates (25%) of blindness, due to glaucoma, have been previously reported in Nepal (20), but much lower rates (7.1-10.8%) have been reported in other south central Asian countries (12, 13). Further research into, for example, the genetics of glaucoma in this unique population may help elucidate the reason for the differences in these findings. Given the high rate of undiagnosed glaucoma we have identified, priority needs to be placed on developing enhanced screening methods in this population as early detection and treatment of POAG can prevent disease progression to visual impairment and blindness.

In conclusion, the prevalence of glaucoma in the Jirel ethnic group of Nepal is high (approximately 4.2% in those 40 years of age or older), and 12.8% of all glaucoma cases are at least unilaterally blind. Moreover, the rate of undiagnosed glaucoma is approximately 94%. Therefore, glaucoma is a major ocular health concern in the Jirel population. Improved screening techniques, such as portable technologies, and increased awareness can help minimize glaucoma-associated burden in Nepal.

## Data Availability Statement

The data presented in the study are deposited in the Center for Open Science, accession number osf.io/cxt7b.

## Ethics Statement

The studies involving human participants were reviewed and approved by University of Texas Rio Grande Valley Institutional Review Board and the Nepal Health Research Council. The patients/participants provided their written informed consent to participate in this study.

## Author Contributions

SM, NB, and JC contributed to data analysis, interpretation of results, and preparation of the manuscript. MJ, SL, JS, JB, BT, ST, and SW-B contributed to data collection, data management, data analysis, interpretation of results, and preparation of the manuscript. All authors contributed to the article and approved the submitted version.

## Funding

This work was supported by R01 EY024384 to SW-B and MJ and was conducted in part in facilities constructed with support from C06 RR020447.

## Conflict of Interest

The authors declare that the research was conducted in the absence of any commercial or financial relationships that could be construed as a potential conflict of interest.

## Publisher’s Note

All claims expressed in this article are solely those of the authors and do not necessarily represent those of their affiliated organizations, or those of the publisher, the editors and the reviewers. Any product that may be evaluated in this article, or claim that may be made by its manufacturer, is not guaranteed or endorsed by the publisher.
